# Identification of Quantitative Trait Loci and Candidate Genes Controlling Seed Dormancy in Eggplant (*Solanum melongena* L.)

**DOI:** 10.3390/genes15040415

**Published:** 2024-03-26

**Authors:** Jiaqi Ai, Wuhong Wang, Tianhua Hu, Haijiao Hu, Jinglei Wang, Yaqin Yan, Hongtao Pang, Yong Wang, Chonglai Bao, Qingzhen Wei

**Affiliations:** 1 Zhejiang Academy of Agricultural Sciences, Hangzhou 310021, China; ajq@stu.zafu.edu.cn (J.A.); wangwh@zaas.ac.cn (W.W.); huth@zaas.ac.cn (T.H.); huhj@zaas.ac.cn (H.H.); wangjinglei@zaas.ac.cn (J.W.); yanyq@zaas.ac.cn (Y.Y.); panghongtao@stu.zafu.edu.cn (H.P.); baocl@zaas.ac.cn (C.B.); 2 College of Horticulture Science, Zhejiang Agriculture and Forestry University, Hangzhou 310021, China; 3 Zhumadian Academy of Agricultural Sciences, Zhumadian 463000, China; wyong2299@163.com

**Keywords:** *Solanum melongena* L., seed dormancy, QTL-Seq, candidate gene

## Abstract

Seed dormancy is a life adaptation trait exhibited by plants in response to environmental changes during their growth and development. The dormancy of commercial seeds is the key factor affecting seed quality. Eggplant seed dormancy is controlled by quantitative trait loci (QTLs), but reliable QTLs related to eggplant dormancy are still lacking. In this study, F_2_ populations obtained through the hybridization of paternally inbred lines with significant differences in dormancy were used to detect regulatory sites of dormancy in eggplant seeds. Three QTLs (*dr1.1*, *dr2.1*, and *dr6.1*) related to seed dormancy were detected on three chromosomes of eggplant using the QTL-Seq technique. By combining nonsynonymous sites within the candidate regions and gene functional annotation analysis, nine candidate genes were selected from three QTL candidate regions. According to the germination results on the eighth day, the male parent was not dormant, but the female parent was dormant. Quantitative real-time polymerase chain reaction (qRT-PCR) was used to verify the expression of nine candidate genes, and the *Smechr0201082* gene showed roughly the same trend as that in the phenotypic data. We proposed *Smechr0201082* as the potential key gene involved in regulating the dormancy of eggplant seeds. The results of seed experiments with different concentrations of gibberellin A_3_ (GA_3_) showed that, within a certain range, the higher the gibberellin concentration, the earlier the emergence and the higher the germination rate. However, higher concentrations of GA_3_ may have potential effects on eggplant seedlings. We suggest the use of GA_3_ at a concentration of 200–250 mg·L^−1^ to treat dormant seeds. This study provides a foundation for the further exploration of genes related to the regulation of seed dormancy and the elucidation of the molecular mechanism of eggplant seed dormancy and germination.

## 1. Introduction

Eggplant (*Solanum melongena* L.) is an important solanaceous vegetable that is rich in vitamins, trace elements, alkaloids, and other nutrients. According to the FAO (https://www.fao.org/home/zh, accessed on 13 March 2024), the total eggplant planting area was 1.846 million hectares worldwide, whereas China accounted for 42.2%. The global production of eggplant was 56.302 million tons, of which China accounted for 65.62%. China is the world’s largest producer, consumer, and exporter of eggplant, which plays an important role in the structure of China’s agricultural industry. *Solanum* species are mainly propagated through seeds. The ability of seeds to germinate rapidly at the same time after sowing is a beneficial trait for producers. In the process of eggplant breeding, the problems of dormancy and the low seed germination rate/uniformity in eggplant varieties or inbred lines are often encountered [1]. Seed dormancy refers to the phenomenon in which viable seeds do not germinate under suitable conditions. Seed dormancy properties are mainly controlled by the maternal parent, and maternal signals control seed properties through a variety of mechanisms [2]. Seeds maintain a static state through dormancy to reduce the risk of being harmed by harsh environments, and high seed vitality is maintained to find the optimal germination time for individual survival [3]. However, seed dormancy is a time-consuming start when screening germplasm resources, affecting the uniformity of seed germination and breeding periods of eggplant. Most commercial eggplant varieties cannot break the dormancy, which may result in a low and uneven seedling germination rate, especially during plug tray cultivation, which seriously affects subsequent planting and field work. These problems further affect breeding programs and have a significant economic impact on seed industries, which need to improve their products’ performance while optimizing the use of economic resources. Accelerated germination treatment of dormant seeds seriously affects the cultivation of high-quality seedlings, which also increases the time and cost. Therefore, the identification of the genetic loci controlling seed dormancy, the mining of candidate genes related to seed dormancy, and the exploration of the mechanisms of candidate genes regulating seed dormancy traits are helpful for cultivating eggplant varieties with both high efficiency and high quality.

Seed dormancy is a quantitative trait controlled by multiple genes, but it is deeply affected by environmental conditions [4]. Studies related to QTLs for seed dormancy have been reported in Arabidopsis, rice, tomato, barley, and other crop species. Jiang et al. [5] identified QTLs controlling seed dormancy using a recombinant inbred line (RIL) population and a two-chromosome segment replacement line (CSSL) population. Liu et al. [6] found two regulatory genes related to seed dormancy in *A. thaliana*: *ODR1* and *bHLH57*. Wheat *TaSdr* obtained from rice *OsSdr4* with a seed dormancy function had significant effects on seed dormancy through the missense mutation SNP643 [7]. *TaQsd1* is also required for the early germination of barley seeds [8]. *DELAY OF GERMINATION-1* (*DOG1*) is a quantitative trait gene regulating seed dormancy; it mainly cooperates with ABA to delay seed germination, and it is a key gene causing dormancy in Arabidopsis plants [9,10]. *DOG1* was found to be essential for inducing seed dormancy when isolated from *A. thaliana* through a combination of map-based cloning and mutant analysis [11]. Zha et al. [12] found that the circadian evening complex (EC) protein LUX ARRHYTHMO (LUX) coordinated with the chromatin remodeling factor PICKLE (PKL) to regulate the expression of the downstream gene *DOG1*. *DOG1* also regulates seed dormancy and germination in *A. thaliana* at a low temperature [13]. *TaDOG1* induces seed dormancy through the abscisic acid 1 (ABA1)-dependent inhibition of hypersensitive germination 1 [14].

At present, there are few studies on eggplant seed dormancy and germination at the DNA level, and no candidate genes in eggplant seeds have been identified through genetic mapping. However, there have been reports of dormancy and germination using eggplant seeds. Zhang et al. [15] studied the effect of a KNO_3_ priming solution mixed with salicylic acid (SA) on the germination and emergence of eggplant seeds at 15 °C. Muthusamy et al. [16] used helium–neon laser irradiation to irradiate eggplant seeds and found that a low dose of He–Ne laser irradiation enhanced the germination process and altered the seeds’ growth. Gonzalez (2015) [17] determined the effects of different concentrations of vinegar on the germination of eggplant seeds. Ali et al. [18] suggested that aqueous garlic (Allium sativum), SA, and methyl jasmonate (MeJA) can promote seed germination and seedling growth in eggplant. Subramaniam [19] identified some candidate genes related to dormancy traits by comparing the FT/TFL1 gene family in terms of seed dormancy using multiple genomes.

In this study, we generated isolated F_2_ populations through the hybridization of eggplant parents with different levels of dormancy and analyzed seed dormancy QTLs at the whole-genome level. Three QTLs (*dr1.1*, *dr2.1*, and *dr6.1*) related to seed dormancy were identified through QTL-Seq while using the latest eggplant reference genome. After combining these results with those of a functional annotation and quantitative analysis of the candidate genes, nine genes that may be involved in regulating eggplant seed dormancy were predicted, and three genes were finally screened. *Smechr0201082* was the most likely gene to regulate the dormancy of eggplant seeds, according to the analysis of the expression profiles and germination data.

## 2. Materials and Methods

### 2.1. Test Materials and Phenotypic Identification

F_1_ was obtained by crossing a male (P_1_-73) with a female (P_2_-22) homozygous inbred line with a substantial difference in dormancy. Both parents were important breeding lines of purple-red and long eggplants. The F_2_ population was obtained through F_1_ self-crossing, in which the maternal parent had strong dormancy characteristics and the male parent had a good germination rate without dormancy characteristics. The parents and F_1_ generation were planted in the Qiaosi experimental field of the Zhejiang Academy of Agricultural Sciences in the spring of 2022. Twenty plants of each parent were planted; ten thereof were used for self-breeding, and the other ten were used for crossbreeding to harvest F_1_. Thirty F_1_ plants were planted. To reduce the influence of seed maturity on seed dormancy, all self-crossed and crossed plants with fruit appearing on the second branch of a single plant were selected for retention. Each fruit was harvested 60 days after pollination. The harvested seeds were placed in a plastic greenhouse for later ripening. When the eggplant fruit became soft, it was washed to separate the seeds from the pulp. A portion of the seeds were then used in the following experiments, and the remainder was stored in seed bags. Three individual seeds of the parents and F_1_ plants were selected for the experiments with three replicates. Ten F_1_ self-crossing seeds were randomly selected, and F_2_ was set for 10 replicates. For each replicate, 100 excellent seeds with normal color and plumpness were selected. The seeds were evenly divided into germinating boxes with filter paper and placed in a variable temperature germinating box (LC-QHX-150BE, Shanghai, China) for a germination test. The germination conditions were as follows: under dark conditions with a relative humidity of 50%, with a cycle of 12 h at 20 °C and 12 h at 30 °C as a cycle. The germination of the seeds was determined every 24 h for 14 consecutive days. In this study, the presence of white bud spots on the seeds was a criterion for germination, and the germination rate on the 8th day was used to evaluate the seeds’ dormancy.

### 2.2. DNA Extraction and Mixing Pool Sequencing

According to the statistical data on the dormancy phenotype, a single F_2_ plant with extreme traits was selected for sampling. The seed coat developed from the integument of the ovule in the ovary of the mother, and the genotype was the same as that of the mother. Therefore, when sampling, the eggplant seed skin needed to be peeled. For F_2_, there were 100 seeds in each replicate and a total of 10 replicates. To ensure the accuracy of the mixed pool results, we selected 3 seeds from each group that germinated at the earliest and the latest and mixed them to form a single plant pool for extreme traits. The number of individual plants sampled was 30, accounting for only 3.0% of the total population. The individual plants were frozen in liquid nitrogen and stored in an ultralow-temperature refrigerator at −80 °C for later use. The DNA of extreme individual strains was extracted using the modified CTAB method, and the DNA quality was assessed with agarose gel electrophoresis. After the concentration was determined with a NanoDrop, the same amount of DNA was mixed into the nondormancy pool I and the dormancy pool (L). The parents and DNA pool were sequenced using the Illumina NovaSeq sequencing technology platform.

### 2.3. Analysis of Seed-Dormancy-Related QTLs

The eggplant genome was assembled using the last three generations of sequencing, and this was used as the reference genome [20]. BAM files were obtained by aligning the clean data with a reference genome sequence using the BWA software [21]. The GATK v4.2.4.1 software was used to correct the BAM files to improve the accuracy of SNP and indel markers that were obtained through detection [22]. GATK’s Haplotyper method was used for indel detection and filtering to obtain the final SNP site set. First, SNP loci with multiple genotypes were filtered out; second, SNP loci with read support of less than 4 were filtered out; third, SNP loci with the same genotypes between the mixed pools and those with recessive mixed pool genes that were not from recessive parents were filtered out to obtain high-quality, trusted SNP loci. The SNP index was calculated according to the QTL-Seq method; the sliding window was set to 2 Mb, and SNP labeling was performed [23]. The SNPEff v5.0e software [24] and gene sequencing information of the reference genome were used for the functional annotation of SNPs and indel mutations, and further SNPs and indels were used for a character mapping analysis. Based on the results of the above mutation detection process, genetic markers of homozygous differences between the two parents (except the F_1_ population) were screened, and SNP markers and indel markers were selected.

### 2.4. Candidate Gene Prediction

The obtained QTL sites related to eggplant seed dormancy were screened for the prediction of seed dormancy controlling genes. Queries for homologous gene sequences from the NCBI (https://www.ncbi.nlm.nih.gov/, accessed on 16 May 2023) and the eggplant genome website (http://eggplant-hq.cn/Eggplant/home/index, accessed on 3 June 2023) were used to compare related genes and annotate the gene functions. Firstly, in the three candidate intervals, the genes with the functions related to seed dormancy were selected according to gene annotation. Secondly, based on previous studies, the sequences of the genes related to seed dormancy in rice, wheat, and tomato were downloaded and blasted against the eggplant genome to detect homologous genes in eggplant. The genes with an identity of over 85% and a coverage over 90% in the QTL regions were also regarded as candidate genes for eggplant seed dormancy. After combining these results with those of the non-synonymous mutation sites, a total of 9 candidate genes were finally selected.

### 2.5. Analysis of Candidate Genes’ Expression Levels

To analyze the relative expression levels of the related genes, eggplant seed samples were collected from parents at different times (0 d, 2 d, 4 d, and 6 d). Total RNA was extracted from the parent seeds with the SYBR GREEN dye method. The mass and concentration of the total RNA were determined with an ND-5000. RNA (300 ng) was added to 12 μL of RNase-free ddH_2_O, and 3 μL of 5× gDNA digester mix was added to form a mixture. A pipette was used to gently blow and mix. After incubation at 42 °C for 2 min, 5 μL of 4× Hifair III SuperMix plus was added to obtain a reaction system with a volume of 20 μL for PCR. The PCR program was set to 25 °C for 5 min, 55 °C for 15 min, and 85 °C for 5 min, and the product was cDNA. BIO-GENER was used for qRT-PCR; the amplification conditions were 95 °C for 5 min with one cycle. A total of 40 cycles were performed at 95 °C for 10 s and 60 °C for 30 s. To calculate the mean and standard deviation, three biological replicates were set for each response, and *TRX* was used as the internal reference gene. The list of gene primers is shown in Table S6.

### 2.6. Treatment of Seeds with Different Concentrations of GA_3_

A total of 1 g of GA_3_ was dissolved in 1.5 mL of ethanol and diluted to 1000 mL with water to be used as a mother liquor. Then, 100, 95, 90, 85, 80, 75, and 70 mL of distilled water were added to 0, 5, 10, 15, 20, 25, and 30 mL of the mother liquor and diluted to concentrations of 0 (control), 50, 100, 150, 200, 250, and 300 mg·L^−1^, respectively. Plummy F_1_ seeds were selected, divided into 7 groups of 100 seeds per group, and placed in mesh gauze bags. Three replicates were set. They were then disinfected with 75% ethanol and washed three to five times with distilled water. Seven GA_3_ solutions with different concentrations were poured into the germination box, and the washed seeds were also placed therein. The seeds were soaked for 12 h in the germination box with various concentrations of GA_3_ solutions. The seeds were removed and washed three times with clean water after 12 h. Then, 21 germination boxes were prepared with 2 sheets of filter paper, followed by wetting of the filter paper with distilled water (with no water flow). The cleaned seeds were placed in the germination box in a 10 × 10 arrangement and then placed in a constant-temperature incubator at 30 °C. The seeds’ germination was observed and recorded at the same time each day.

## 3. Results

### 3.1. Statistical Analysis of the Seed Dormancy Characteristics

**Figure 1:**
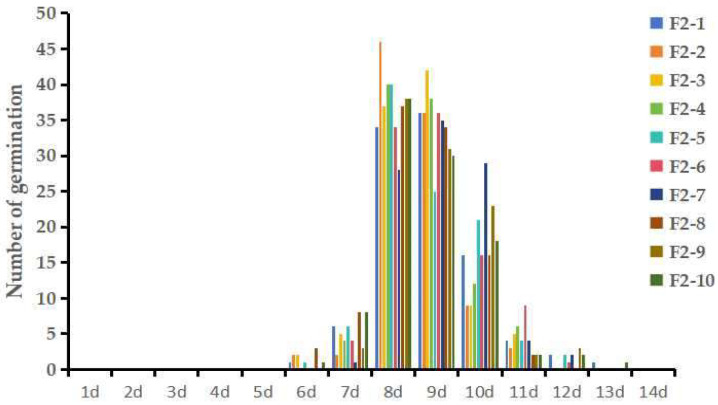
Daily germination of the 10 replicates in the F_2_ population.

The germination rates of the parents (P_1_-73 and P_2_-22), F_1_, and F_2_ at 1–14 d are shown in Table 1. The dormancy phenotype of the eggplant seeds was represented by the germination rate on the eighth day. The seed dormancy of the eggplant parents was quite different; the male parents began to germinate on the fourth day, and the germination rates of the male seeds were 94.3% on the eighth day and 100% on the eleventh day. However, the female seeds began to germinate on the seventh day, and the germination rate was only 14% on the eighth day and 76.3% on the fourteenth day. These statistics indicated that the male seed dormancy was weak, while the female seed dormancy was strong. The F_1_ populations began to germinate on the sixth day, and the germination rates were 14.3% on the eighth day and 84.3% on the fourteenth day, so the F_1_ seeds also had strong dormancy characteristics. Among the 10 replicates of the 1000 F_2_ seeds collected, the average germination rates were 42.9% on the eighth day and 99.6% on the fourteenth day. As shown by the statistical data on the daily germination of the F_2_ seeds, their dormancy was normally distributed, indicating that the dormancy of eggplant seeds is a quantitative trait that is controlled by multiple genes (Figure 1; Table S1).

### 3.2. Parental and Extreme Mixed Pool Sequencing

A total of 96 G of clean data were generated through Illumina sequencing, and the value of Q30 reached 92.14%, indicating that the sequencing data were of a high quality. The average sequencing depth of the parents was 9.77×, and that of the mixed pools was 19.45×. The average efficiency of the comparison between the samples and the reference genome was 94.34%, while the average coverage depth and the genome coverage were 22× and 89.78% (coverage of at least one base), respectively, which guaranteed the accuracy of the BSA analysis. The sequencing quality of the parents and extreme cells satisfied the requirements of the QTL-Seq analysis. Using the latest eggplant reference genome, a total of 1,078,479 SNPs and 1,078,479 indels were detected (Table 2).

### 3.3. Detection of QTL Sites with Major Effects

**Figure 2:**
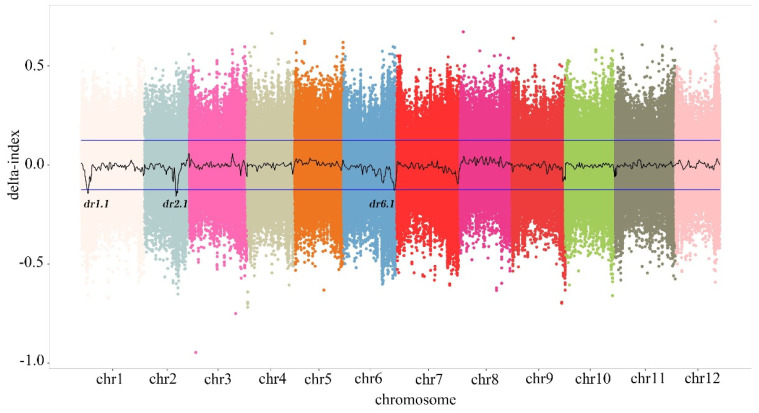
The △ (SNP index) distribution of the QTLs for the seed dormancy index detected with QTL-Seq. Chromosome distribution of the SNP/indel indexes in two progenies.

Three QTL candidate regions related to eggplant dormancy were detected using QTL-Seq analysis of the parent and extreme pool data, and they were mainly distributed on three eggplant chromosomes: chromosome 1 (chr1), chromosome 2 (chr2), and chromosome 6 (chr6). The three QTLs were named *dr1.1*, *dr2.1*, and *dr6.1*. *dr1.1* was located in the 10.295–12.763 Mb region of chr1, while *dr2.1* was located in the 52.221–55.671 Mb region of chr2, and *dr6.1* was located in the 85.328–85.987 Mb region of chr6 (Figure 2, Table 3).

### 3.4. Candidate Gene Prediction

**Figure 3:**
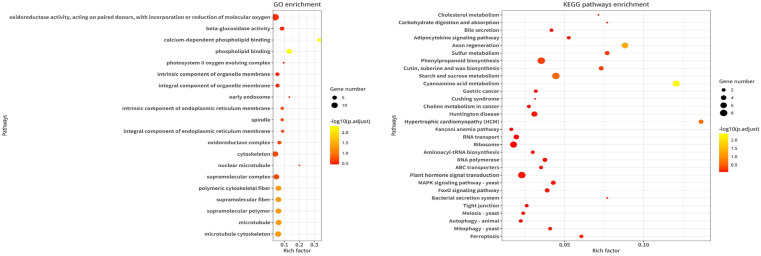
GO and KEGG enrichment analysis.

There were 300 nonsynonymous mutation sites in the three candidate regions identified for the QTL sites (Table S2). Totals of 38, 13, and 17 genes associated with effective mutations were found in the candidate regions of chr1, chr2, and chr6, respectively (Figure 3; Table S3). In addition, a total of 11 genes related to seed dormancy within or near the candidate regions were found in eggplant after a comparison of the homology with *A. thaliana* and rice (Table S4). In conclusion, 79 candidate genes that could be associated with eggplant seed dormancy were identified. According to the data provided by the effective mutation association gene annotation with the SNP index and effective mutation association, nine candidate genes were finally screened, namely, *Smechr0101047*, *Smechr0101084*, *Smechr0101118*, *Smechr0101241*, *Smechr0101299*, *Smechr0201082*, *Smechr0201292*, *Smechr0602743*, and *Smechr0602744*.

### 3.5. Verification of Quantitative Expression

**Figure 4:**
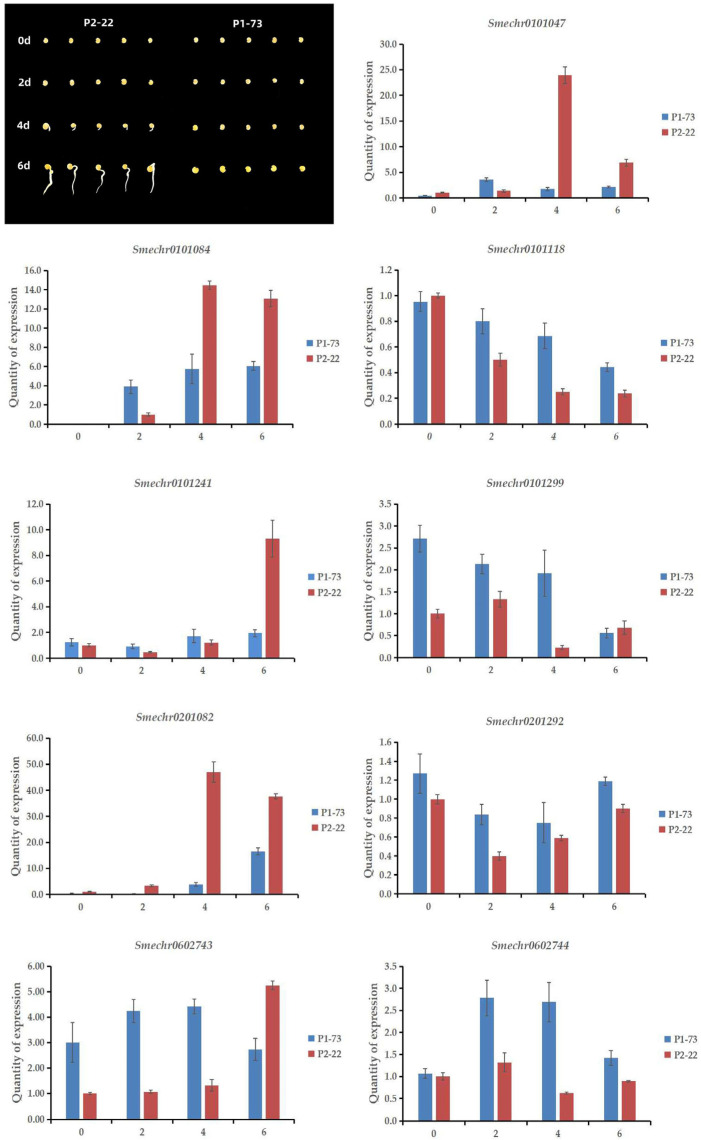
Analysis of the two test materials (P_1_-73 and P_2_-22) when used as seed samples at different times (0, 2, 4, 6 d) and the qRT-PCR results for the expression of genes related to seed dormancy. The relative expression levels were analyzed at 0, 2, 4, and 6 d.

To identify the main gene, qRT-PCR was used to verify the expression of the nine predicted genes related to eggplant dormancy in the parents. The paternal parent at 0 d was used as the control. Because *Smechr0101084* was not expressed in the paternal sample at 0 d, the control of this gene was changed to the paternal parent at 2 d (Figure 4). According to the results, the expression levels of *Smechr0101047* in both parents were relatively low at 0 d and 2 d; the expression level in the male parent was the highest at 4 d, and its expression levels at 0 d, 4 d, and 6 d were higher than those of the female parent. The expression of the female parent was the highest on the second day. *Smechr0101084* was not expressed in either parent at 0 d, and the expression level in the male parent was higher than that in the female parent at 4 d and 6 d. Moreover, the expression level of *Smechr0101084* in the female parent was higher than that in the male parent at 2 d and increased gradually with time. The expression levels of *Smechr0201082* in the two parents were very low at 0 d and 2 d, whereas the expression was higher in the male parent than in the female parent, especially at 4 d. The expression of *Smechr0201082* in the female parent gradually increased but was always lower than that in the male parent. *Smechr0101118*, *Smechr0101299*, *Smechr0201292*, *Smechr0602743*, and *Smechr0602744* began to be expressed at 0 d in the two parents but were expressed at low levels from that day. The expression of *Smechr0101241* in the female parent was consistently low, whereas the expression in the male parent was the highest at 6 d. According to the statistical data on the trait of dormancy, the male and female seeds began to germinate at 4 d and 7 d, respectively. The germination rate of males was higher than that of females on the 14th day. Therefore, we concluded that the expression of the *Smechr0101047*, *Smechr0101084* and *Smechr0201082* genes at 4 and 6 d was consistent with the germination trend of the parents at 4, 6, and 14 d. However, according to the comparison with the expression trends of the other two genes, *Smechr0201082* was the most likely candidate gene regulating seed dormancy.

### 3.6. Effects of Different Concentrations of GA_3_ on the Germination Percentage

**Figure 5:**
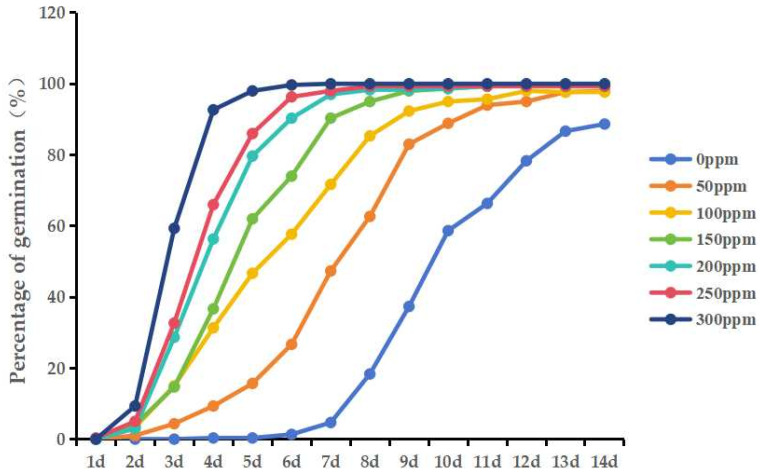
Germination rate (%) of seeds after treatment with different GA_3_ concentrations.

In the control treatment, the seeds germinated from the fourth day, and the germination rate gradually increased from 6 to 12 d, reaching 88.67% on the fourteenth day. When the concentration of GA_3_ was 50 mg·L^−1^, the germination rate was less than 30% from 0 to 6 d, and it significantly increased from 7 to 10 d. The germination rate reached 95% or more on the twelfth day. The germination rate of seeds treated with GA_3_ at a concentration of 100 mg·L^−1^ was only about 15% from 0 to 3 d, and it reached 85% or more from 4 to 8 d. The germination rate reached 95% or more on the tenth day. The germination rates of seeds treated with GA_3_ at concentrations of 150, 200, and 250 mg·L^−1^ were higher from the second day. The germination rate of the seeds treated with 150 mg·L^−1^ GA_3_ reached 90% or more on the seventh day, and the germination rate was above 98% on the tenth day. The germination rate of seeds treated with a concentration of 200 mg·L^−1^ was more than 90% on the sixth day, and the germination rate was more than 98% on the eighth day. The germination rate of seeds treated with GA_3_ at a concentration of 250 mg·L^−1^ was more than 95% on the sixth day, and the germination rate was more than 98% on the seventh day. The seeds treated with GA_3_ at a concentration of 300 mg·L^−1^ showed the fastest germination speed, with the germination rate reaching 90% on the fourth day and 100% on the seventh day (Figure 5; Table S5). In conclusion, within a certain range, the GA_3_ concentration was positively correlated with the germination rate, and the time of emergence could also be advanced. In this experiment, the concentration of GA_3_ was 200–250 mg·L^−1^, and the germination rate of the seeds at 6 d was already more than 90%. Therefore, choosing to soak seeds with a shallow dormancy in GA_3_ at a concentration of 200–250 mg·L^−1^ for 12 h before seeding is sufficient to relieve dormancy; too high a concentration of GA_3_ may have effects on the seeds.

## 4. Discussion

Seed dormancy is an important factor and also an effective way to regulate the germination rate. Although seeds with dormancy have better adaptability, there are some problems in breeding practices due to the long germination times and low germination rates. The degree of seed dormancy was negatively correlated with the germination time. In general, germplasm resources that germinate earlier allow the collection of phenotypic data as early as possible, which is also convenient for subsequent breeding work. Therefore, it is important to investigate the dormancy of eggplant seeds in order to breed better eggplant varieties. However, dormancy in eggplant is a complicated process due to the many overlapping and interacting factors affecting the absolute dormancy levels. Seed dormancy is affected by environmental, genetic, physiological, and agronomic factors, including light, temperature, carbon dioxide content, and hormones (i.e., auxin, cytokinin, and gibberellin). The seed dormancy phenotype is inherited maternally [2,25,26]. Previous studies showed that plant hormones play important roles in seed dormancy and germination [27,28]. In the variable external environment, seeds also frequently occur with other stimuli, such as those of water potential, nitrate, and other environment-related signals, whereas the mechanisms of these signals in relation to seed dormancy and germination are still not fully understood [29].

There are probably multiple QTL sites for eggplant seed dormancy that are related to eggplant domestication or evolution and may play roles in the dormancy of different subspecies or varieties. These factors represent a great challenge in the mining of QTLs. To identify genes related to seed dormancy in eggplants, we used parents with significant differences in seed dormancy during breeding to construct isolated populations. Three QTL sites related to seed dormancy were detected with a QTL-Seq analysis, namely, *dr1.1*, *dr2.1*, and *dr6.1*. They were located at 10.295–12.762 Mb on chromosome 1 (*dr1.1*), 52.220–55.670 Mb on chromosome 2 (*dr2.1*), and 85.328–85.987 Mb on chromosome 6 (*dr6.1*), respectively. Nine candidate genes were selected through the analysis of nonsynonymous mutation sites and gene functional annotation. To narrow the range and identify the genes that were most closely related to eggplant seed dormancy, qRT-PCR was used to evaluate the expression levels of these nine candidate genes during seed dormancy and germination. Based on the germination rates of the eggplant seed parents and F_1_, we analyzed the quantitative expression results with qRT-PCR and screened out three candidate genes, *Smechr0101047*, *Smechr0101084*, and *Smechr0201082*, which were consistent with the trait of seed dormancy in eggplant. Melan et al. [30] found that the *LOX1* gene was temporarily expressed in the epidermal and aleurone layers of Arabidopsis seedlings during germination to regulate dormancy and germination. In the present study, we identified the gene *Smechr0101047*, which was homologous to the dormancy-related gene *LOX1* in Arabidopsis, through sequence comparison. These genes may share similar functions in regulating seed dormancy. In rice, *SD6* and *ICE2* indirectly regulate the key ABA synthesis regulatory gene *NCED2* by antagonizing the regulatory transcription factor *OsbHLH048*, thus achieving timely and efficient regulation of ABA content so that seeds can switch from dormancy and germination at any time [31]. The *OsbHLH048* transcription factor was found to be homologous to the Smechr0101084 gene identified in the present study. *Smechr0201082*, a *WRKY72* gene associated with GA and/or ABA signaling, was screened in the region of chromosome 2 [32]. According to the phenotypic data shown in Table 1, P_1_ and P_2_ appeared to be white at 7 and 4 d, respectively. According to the results of qRT-PCR, *Smechr0201082* was considered to be the most likely candidate gene for regulating the dormancy of eggplant seeds. *Smechr0201082* caused seed germination to begin before 4 d; the seeds received a signal to gradually germinate, and a white color bud spot began to appear at 4 d. The germination rate of seeds without dormancy was higher than that of seeds with dormancy.

Seed maturation, germination, and dormancy may be regulated by interactions between plant hormone and transcription factor networks. Recent findings have revealed that WRKYs—especially those mediated by GA and/or ABA—play a multifaceted role in seed dormancy and germination [33,34]. *OsWRKY51* and *OsWRKY71* are key regulatory factors mediating the interaction between GA and ABA in rice aleurone cells and embryos [35,36]. Xie et al. [37] verified that the rice gene *OsWRKY72* was induced by ABA. *AtWRKY27* from Arabidopsis is directly regulated by the GA signaling component RGA and is involved in GA-mediated seed germination [38]. *OsWRKY29*, a new ABA signaling repressor, inhibits seed dormancy by directly down-regulating the expression of *OsABF1* and *OsVP1* [39]. Wang et al. [40] found that *WRKY72* could restrict the inhibitory effect of GA on rice seed germination. Among these hormones, ABA and GA had antagonistic effects on seed dormancy and germination. ABA inhibited germination and promoted dormancy, whereas GA broke dormancy and promoted germination. In the last few decades, the role of ABA and GA biosynthesis and signal transduction in controlling seed dormancy and germination has been extensively studied [40,41,42,43]. For example, in the Arabidopsis PEBP gene family, the MFT gene promotes seed germination and breaks seed dormancy by interacting with the GA and ABA signaling pathways [44]. In the future, we will verify the specific function of *Smechr0201082* in the process of seed dormancy and germination, and we will further clarify the regulation of the hormone signaling pathways and the regulatory networks of gene and hormone signaling. In addition, we will develop molecular markers to assist in the screening of eggplant germplasm resources based on mutation sites. In the breeding process, germplasm resources with dormancy characteristics should be avoided as maternal parents. This is because the degree of seed dormancy at abscission is genetically determined, and the environmental conditions experienced by the maternal parent significantly affect the seed properties [25,26]. Therefore, when selecting parents for eggplant breeding, the germplasm resources with see dormancy are not appropriate as the maternal parent. When excellent germplasm resources with dormancy are selected as the parent, GA_3_ can be used at a concentration of 200–250 mg·L^−1^ to soak the seeds for 12 h in advance, as this can effectively relieve the seeds’ dormancy, shorten the dew time, and improve the germination rate. In summary, *Smechr0201082* may regulate the synthesis of plant hormones, and it can affect the ability of seeds to sense the surrounding environment together with the homeostasis of catabolism, so as to control the dormancy or germination of eggplant seeds. However, the molecular mechanism by which *Smechr0201082* regulates the eggplant seed dormancy or germination process remains to be further investigated.

## 5. Conclusions

In this study, three major QTLs related to seed dormancy were detected using QTL-Seq technology, and potential candidate genes related to the regulation of eggplant dormancy were further identified with gene annotation analysis and qRT-PCR. The results indicated that the expression of the *Smechr0201082* gene mediated by GA and/or ABA was more consistent with the dormancy phenotype of the parent seeds. In addition, it was experimentally determined that, with a certain range, the higher the concentration of GA, the higher the germination rate. However, in the breeding process, keeping the GA_3_ concentration in the range of 200–250 mg·L^−1^ can not only ensure the quality of eggplant seedlings but also reduce their cost. Briefly, the present study lays a foundation for the further exploration of seed dormancy genes in eggplant and the analysis of their molecular mechanisms, in addition to providing a referential basis for agricultural practices in eggplant breeding and cultivation.

## Figures and Tables

**Table 1:** Statistical data on the germination rates of the parents, F_1_, and F_2_. The first row of the table refers to the germination days. The seed germination percentage (%) was assessed on days 1 to 14. The first column presents the test materials, and the other columns present the germination rates of the test materials on the corresponding days.

Test Material	1 d	2 d	3 d	4 d	5 d	6 d	7 d	8 d	9 d	10 d	11 d	12 d	13 d	14 d
P_1_-73	0.0%	0.0%	0.0%	0.0%	0.0%	0.0%	5.3%	14.0%	33.0%	52.0%	63.3%	67.3%	72.3%	76.3%
P_2_-22	0.0%	0.0%	0.0%	2.3%	15.7%	38.0%	75.7%	94.3%	98.3%	98.7%	100.0%	100.0%	100.0%	100.0%
F_1_	0.0%	0.0%	0.0%	0.0%	0.0%	2.0%	3.3%	14.3%	32.0%	44.7%	56.0%	69.7%	80.7%	84.3%
F_2_	0.0%	0.0%	0.0%	0.0%	0.0%	1.0%	5.7%	42.9%	77.2%	94.1%	98.2%	99.4%	99.6%	99.6%

**Table 2:** Statistics of the sequencing data for the two parents and extreme pools.

Sample ID	Raw Reads	Raw Bases	Q30 (%)	GC (%)	Mapped (%)	Genome Coverage 1× (%)	Average Depth	SNP Number	Indel Number
P1-73	83,794,760	12,653,008,760	91.48	37.9758	93.99	89.13	10.09	685,891	117,150
P2-22	77,173,102	11,653,138,402	91.46	37.3624	95.53	89.16	9.45	521,259	96,169
E-pool	252,479,026	38,124,332,926	91.55	38.1669	93.46	90.45	30.22	912,593	196,108
L-pool	232,529,434	35,111,944,534	91.58	37.9924	94.40	90.40	28.05	909,687	191,146

**Table 3:** Summary of the QTLs detected for the seed dormancy index with QTL-Seq.

QTL	Chromosome	Start/Mb	End/Mb	Interval/Mb	No. of Genes Associated with Effective Mutations
*dr1.1*	chr1	10.295	12.763	2.468	38
*dr2.1*	chr2	52.221	55.671	3.450	13
*dr6.1*	chr6	85.328	85.987	0.659	17

## Data Availability

The data supporting the findings of this study are available from the corresponding author upon reasonable request.

## Supplementary Materials

The following supporting information can be downloaded at https://www.mdpi.com/article/10.3390/genes15040415/s1. Table S1: Phenotypic data of parents, F_1_ and F_2_ population; Table S2: SNP-index data statistics of QTL candidate regions; Table S3: Effective mutation-associated genes; Table S4: The genes related to seed dormancy were obtained by homologous comparison; Table S5: Number of seeds germinated at different concentrations of GA_3_; Table S6: Seed dormancy related candidate genes and their primers.
